# Prevalence and predictors of treatment-resistant schizophrenia in a tertiary hospital in Northeast Brazil

**DOI:** 10.47626/2237-6089-2020-0151

**Published:** 2021-12-10

**Authors:** Douglas de Sousa Soares, Danyelle Rolim Carvalho, Mellanie Dellylah Trinta Ribeiro, Elton Jorge Bessa Diniz, Alcides Ferreira Rêgo

**Affiliations:** 1 Programa de Residência Médica em Psiquiatria Hospital de Saúde Mental Professor Frota Pinto Fortaleza CE Brazil Programa de Residência Médica em Psiquiatria, Hospital de Saúde Mental Professor Frota Pinto, Fortaleza, CE, Brazil.; 2 Programa de Esquizofrenia Departamento de Psiquiatria Universidade Federal de São Paulo São Paulo SP Brazil Programa de Esquizofrenia (PROESQ), Departamento de Psiquiatria, Universidade Federal de São Paulo (UNIFESP), São Paulo, SP, Brazil.; 3 Laboratório Interdisciplinar de Neurociências Clínicas Departamento de Psiquiatria UNIFESP São Paulo SP Brazil Laboratório Interdisciplinar de Neurociências Clínicas (LiNC), Departamento de Psiquiatria, UNIFESP, São Paulo, SP, Brazil.

**Keywords:** Treatment-resistant schizophrenia, clozapine, schizophrenia, psychosis, epidemiology

## Abstract

**Objective:**

To investigate epidemiological factors related to treatment-resistant schizophrenia (TRS) in Northeast Brazil, a region where data about mental health are still scarce.

**Methods:**

This retrospective cross-sectional study included all patients with schizophrenia currently receiving treatment at the outpatient psychiatric clinic of a tertiary hospital in Northeast Brazil. They were divided into TRS and treatment-responsive groups, and epidemiological characteristics of both groups were compared. A logistic regression model investigated factors related to treatment resistance.

**Results:**

Two hundred and five patients were included, 155 treatment-resistant and 50 treatment-responsive. The TRS group had higher use of benzodiazepines (36.1 vs. 18%, p = 0.017) and antiepileptics (36.8 vs. 8.0%, p < 0.001), antipsychotic polypharmacy (28.6 vs. 8%, p = 0.003) and suicide attempts (35.6 vs. 20%, p = 0.04). Age at onset was younger (19.7±7.3 vs. 24.6±8.6 years, p = 0.001) and CGI was higher in TRS (3.72±1.00 vs. 3.16±1.00, p = 0.001). In logistic regression, being married was a protector (odds ratio [OR] = 0.248, 95% confidence interval [95%CI] 0.091-0.679, p = 0.007) and younger age at onset was a predictor (OR = 1.076, 95%CI 1.034-1.120, p < 0.001) of treatment resistance.

**Conclusion:**

Early onset of disease was associated with more treatment resistance, while being married with less resistance. Clinicians should identify early predictors of resistance in order to reduce unfavorable outcomes.

## Introduction

Schizophrenia usually presents as a chronic, severe, and treatable mental disorder, with a worldwide prevalence of 0.33 to 0.75%.^1^ It is characterized by a combination of positive, negative, and cognitive symptoms, but may also affect several domains such as thought processes, social interaction, and emotional responsiveness.^2^ Although antipsychotics have been considered the treatment of choice for decades, 20-30% of individuals with schizophrenia do not exhibit adequate response after first- and second-line therapies, being described as having treatment-resistant schizophrenia (TRS).^3^ TRS causes significant economic burden for health systems and intense suffering for affected individuals and their caregivers. Patients with TRS also present elevated rates of comorbidities, iatrogenic side effects and unemployment. Hospitalization costs of TRS were also 10-fold higher than those for general schizophrenia in the United States (US) and 60-fold higher than those for the general U.S. population.^4^

The definition of treatment resistance has been a matter of discussion and several guidelines have used different criteria to establish it, which makes it difficult to compare studies and leads to a lack of high-quality data.^3 , 5^ In order to minimize these divergencies, a panel of specialists from the Treatment Response and Resistance in Psychosis (TRRIP) Working Group published a consensus guideline that defines TRS as < 20% symptom reduction in at least two prospective trials with different antipsychotics at adequate doses lasting more than 6 weeks each.^6^

In the last decades, some studies have investigated epidemiological predictors of TRS, and only a few reviews have been published about this topic.^7 - 9^ This leaves a gap in scientific knowledge, leading to difficult identification of patients at high risk for TRS in clinical practice. According to those studies, predictors of TRS were: longer duration of untreated psychosis (DUP), younger age at disease onset, higher severity of negative symptoms, lower education, hospital admission at diagnosis, lower premorbid functioning, paranoid subtype, substance use, non-adherence to treatment, and suicide attempts.^7 - 9^ It is important to identify individuals at high risk for TRS, since they manifest more impaired cognitive functioning, poorer psychosocial functioning, and, if not adequately treated, potentially more severe psychopathology.^10^

Clozapine, an atypical antipsychotic with unique pharmacological properties, has been used as standard treatment for TRS since Kane et al.^11^ demonstrated its superiority when compared to other antipsychotics. Subsequent studies have evidenced its efficacy in reducing both positive and negative symptoms as well as lowering extrapyramidal syndrome and improving cognitive deficits.^12 , 13^ In addition, clozapine seems to reduce rehospitalization rates, suicide risk and mortality in patients with TRS.^14 , 15^ Despite that, clozapine is licensed in most countries as a third-line therapy, reserved for TRS cases, mainly due to its side effects, especially agranulocytosis and other blood dyscrasias.^16^

Given the social and economic impact of TRS, it is pivotal to describe factors associated with treatment resistance in order to identify TRS cases and initiate prompt treatment. Therefore, the present study aims to compare epidemiological characteristics of TRS and treatment-responsive patients, as well as to identify factors associated with treatment resistance among individuals with schizophrenia receiving treatment at a tertiary mental health hospital in Brazil.

## Methods

### Individuals and study design

This is a retrospective cross-sectional study that included all adult patients with schizophrenia admitted to the psychiatric outpatient clinic of Hospital de Saúde Mental Professor Frota Pinto, in Fortaleza, Brazil, and who were currently attending appointments until December 31, 2019. The hospital is a psychiatric referral center for the entire Northern Region of Brazil, being responsible for the care of the most severe cases of psychiatric conditions in Ceará, a state with a population of about 9 million people. Three independent researchers (DSS, DRC, and MDTR) reviewed and collected data from the records of all patients who were clinically diagnosed with schizophrenia by the team of assistant physicians, based on criteria from the International Classification of Diseases, 10th revision (ICD-10; code F20).

All individuals with ≥ 18 years who attended a psychiatry appointment or were submitted to psychiatric evaluation in the previous 6 months before data collection were included. Patients with diagnoses other than schizophrenia (such as schizoaffective disorder, bipolar disorder, substance-induced psychosis, or persistent delusional disorder), those with evidence of structural brain disease, or who suffered previous severe head trauma with loss of consciousness, were not included. Neither were included patients whose charts were not proper for reading, whose data were not reliable, or patients described as having had comorbid intellectual deficit prior to the first episode of psychosis.

In the present study, the most recent and robust definition for TRS, namely that proposed by the TRRIP Working Group, was used to define treatment resistance.^6^ All treatments with antipsychotics were recorded, including dose and trial duration, and researchers defined as treatment-resistant those patients with two or more ineffective antipsychotic trials at adequate doses for more than 6 weeks each, based on TRRIP guidelines. The Clinical Global Impression (CGI) score was estimated based on information collected by the researchers on the latest medical records. If any divergencies in classifying patients between treatment-resistant or treatment-responsive occurred between the authors, a fourth researcher (AFRN) with more clinical experience was consulted.

### Parameters and definitions

Demographic, clinical, comorbidity and treatment data were collected between February 1st 2019 and December 31st 2019 for subsequent statistical analysis. Epidemiological data included current age and gender, marital status at onset, years of formal education before the first episode of psychosis, age at disease onset, DUP, and duration of disease. Clinical and comorbidity characteristics encompassed hospital admissions, suicide attempts, substance use before the first episode of psychosis, diabetes mellitus, smoking, anxiety, depression, obsessive-compulsive disorder, and the latest CGI score. Finally, treatment parameters included all antipsychotic trials, but also antipsychotic polypharmacy (APP), and current use of other psychotropic drugs such as antidepressants, benzodiazepines, antiepileptics, mood stabilizers, and anticholinergics. APP was defined as the concomitant use of two or more antipsychotics, in antipsychotic doses, except in cases of cross-titration. Patients who were hospitalized for psychiatric reasons three or more times after disease onset were considered having multiple hospital admissions.

### Statistical analysis

Data were entered into Excel spreadsheets and analyzed using SPSS Statistics for Windows version 24.0 (IBM, Chicago, IL, USA). Patients were divided into two groups, namely, patients with TRS, and non-resistant (or treatment-responsive) patients. Demographic and clinical characteristics, comorbidities and treatment information were compared between the two groups. Kolmogorov-Smirnov test was performed for numeric variables, in order to assess variable distribution. Variables with a normal distribution were expressed as mean ± standard deviation. Variables with a non-normal distribution were expressed as median values. Categorical variables were compared using Pearson’s chi-square, while numeric variables were compared using Student’s *t* test (for variables with normal distribution) or Mann-Whitney test (for variables with non-normal distribution). P values ≤ 0.05 were considered statistically significant. To evaluate the factors associated with treatment resistance, a logistic regression model was used. Adjusted odds ratios (ORs) and 95% confidence intervals (95%CIs) were calculated. All variables presenting statistical significance (p ≤ 0.05) in the univariate analysis were included in the logistic regression.

### Ethics

All procedures of this study followed the standards of international ethical recommendations for research with human beings, based on the Declaration of Helsinki (1975), as revised in 2013. Before initiation, the study protocol was analyzed and authorized by the ethics committee of Hospital de Saúde Mental Professor Frota Pinto (CAAE 25911819.8.0000.5047).

## Results

Overall, 205 patients with schizophrenia were included in the study. Demographic characteristics of the TRS and treatment-responsive groups are presented in Table 1 . There were no differences between the groups in terms of age, gender or education level, but the prevalence of individuals who were married prior to the first episode of psychosis was significantly higher in the treatment-responsive group than in patients with TRS (24 vs. 11.6%, p = 0.031). The TRS group also had higher rates of suicide attempts (35.6 vs. 20%, p = 0.04) and multiple hospital admissions (39 vs. 18.40%, p = 0.008).

**Table 1:** Demographic characteristics and disease profile of patients with TRS and treatment-responsive patients

	Treatment-resistant		p

	Yes (n = 155)	No (n = 50)	
Age (years), mean ± SD	37.7±10.6	40.6±12.5	0.112
Gender			
Male	114 (73.5)	40 (80)	0.359
Female	41 (26.5)	10 (20)	
Marital status at onset			
Married	18 (11.6)	12 (24)	0.031
Not married	137 (88.4)	38 (76)	
Education			
< 8 years	89 (57.4)	26 (52)	0.592
> 8 years	66 (42.6)	24 (48)	
First episode			
Yes	2 (1.30)	7 (14)	< 0.001
No	153 (98.7)	43 (86)	
Suicide attempt			
Yes	52/146 (35.6)	10/50 (20.0)	0.040
No	84/146 (64.4)	40/50 (80.0)	
Multiple hospital admissions			
Yes	57/146 (39)	9/49 (18.40)	0.008
No	89/146 (61)	40/49 (81.6)	

SD = standard deviation; TRS = treatment-resistant schizophrenia.

Data presented as n (%), unless otherwise specified.

Pearson’s chi-square and Student’s *t* test were used.

The percentage of patients who were currently smoking was higher among treatment-responsive patients when compared to patients with TRS (22 vs. 11%, p = 0.048). There were no differences between the groups in any other clinical or psychiatric comorbidity variables ( Table S1 , available as online-only supplementary material). When comparing treatment data, the TRS group had higher rates of clozapine use (85.8 vs. 0.00%, p < 0.001) and APP (28.6 vs. 8%, p = 0.003) than the treatment-responsive group. Patients with TRS also received significantly more benzodiazepines (36.1 vs. 18%, p = 0.017) and antiepileptics (36.8 vs. 8.0%, p < 0.001) than treatment-responsive individuals ( Table 2 ).

**Table 2:** Treatment characteristics of patients with TRS and treatment-responsive patients

	Treatment-resistant		p

	Yes (n = 155)	No (n = 50)	
Current clozapine use			
Yes	133 (85.8)	0 (0.00)	< 0.001
No	22 (14.2)	50 (100)	
Previous clozapine use			
Yes	18/154 (11.7)	4/50 (8.0)	0.465
No	136/154 (88.3)	46/50 (92.0)	
LAI antipsychotic			
Yes	18/153 (11.8)	8/48 (16.70)	0.377
No	135/153 (88.2)	40/48 (83.3)	
Treatment interruption			
Yes	33 (21.30)	9 (18)	0.616
No	122 (78.7)	41 (82)	
Antipsychotic polypharmacy			
Yes	44/154 (28.60)	4 (8.0)	0.003
No	110/154 (71.4)	46 (92)	
Medication use			
Antidepressants	78 (50.3)	18 (36)	0.078
Benzodiazepines	56 (36.1)	9 (18)	0.017
Mood stabilizers	4 (2.6)	1 (2.0)	0.827
Antiparkinsonians	23 (14.80)	4 (8.0)	0.214
Antiepileptics	57 (36.8)	4 (8.00)	< 0.001

Data presented as n (%).

LAI = long-acting injectable.

Pearson’s chi-square test was used.

Age at disease onset was significantly lower in the TRS group than in the treatment responsive group (19.7±7.3 vs. 24.6±8.6 years, p = 0.001), while current CGI score was significantly higher (3.72±1.00 vs. 3.16±1.00, p = 0.001). There were no differences between the groups with regard to duration of disease or DUP ( Table 3 ).

**Table 3:** Disease characteristics of patients with TRS and treatment-responsive patients

	Treatment-resistant (n = 155)		Non-resistant (n = 50)		p

	Mean	SD	Mean	SD	
Age at disease onset (years)	19.7	7.3	24.6	8.6	0.001
Duration of disease (years)	17.7	9.0	15.9	10.6	0.226
DUP (months)	16.2	31.2	24.8	51.0	0.303
Current CGI score	3.72	1.00	3.16	1.00	0.001

CGI = Clinical Global Impression; DUP = duration of untreated psychosis; SD = standard deviation.

Student’s *t* test and Mann-Whitney test were used.

The logistic regression model evidenced that being married before disease onset was a protective factor against TRS (OR = 0.248, 95%CI 0.091-0.679, p = 0.007), whereas age at disease onset was a risk factor for TRS (OR = 1.076, 95%CI 1.034-1.120, p < 0.001). Other variables included in the logistic regression did not achieve statistical significance.

## Discussion

The individuals included in the present study received treatment at an outpatient psychiatric clinic of Hospital de Saúde Mental Professor Frota Pinto, i.e., a tertiary mental health hospital, which may explain the elevated rate of patients with TRS in the sample (75.6%). This rate is even higher than the one reported in another study, also conducted in a Brazilian tertiary center, where TRS accounted for 53% of the patients with schizophrenia.^17^ As a referral center, the hospital is responsible for treating and following the most severe and complex cases of schizophrenia in the entire state of Ceará; therefore, it is expected that these severely ill patients will present high treatment resistance rates. Also, in the present study, the sample comprised patients who were mostly in the chronic phase of their disease, with just a few first-episode individuals.

There were no differences between the groups in gender or level of education, but more than half of the sample had attended school for less than 8 years, which is similar to the general mean level of education found in the state of Ceará. This may reflect both a social situation (difficult access to formal education) and a clinical condition (cognitive impairment prior to the first episode of psychosis). Poverty and lack of access to education are unfortunate realities in Brazil, especially for those older than 30-40 years. Poor socioeconomic status and income inequality are moderators of cognitive functioning in patients with schizophrenia and may have an impact on academic performance.^18^

Studies suggest that these social and environmental factors interact with genetic characteristics and influence several outcomes.^18^ Another possible explanation for the low education level is premorbid cognitive impairment. Previous studies have demonstrated that poor premorbid social functioning, including interpersonal relationships, isolation, and social withdrawal, is related to TRS.^19^ This, however, could not be confirmed in the present study.

Interestingly, it was observed that treatment-responsive patients were more frequently married before the first episode of psychosis, and that being married was a protector for TRS. These findings oppose to those of Wimberley et al., who found no association between TRS and living or not with a partner.^8^ However, for patients with schizophrenia, being married may indicate better social support and premorbid functioning.^20^ In Brazil, the burden of caring for patients with schizophrenia resides mostly with the family, and living with a partner may help identify psychotic episodes, increase medication adherence, and improve attendance to doctor’s appointments, which may be linked to better outcomes. Research has demonstrated that living with a partner can predict functional improvement in patients with TRS, corroborating the hypothesis that being married provides additional social support and suggests better functioning.^20^ A Chinese cohort study also demonstrated that married patients with schizophrenia had a caregiver in almost 100% of cases, while only 61-83% of unmarried individuals had one. The same cohort evidenced higher rates of suicide attempts, homelessness and unemployment, more severe psychopathology, poorer mental health status and lower social functioning in the unmarried group.^21^ Finally, being married has also been related to better quality of life and decreased risk for criminal behavior, especially among men.^22 , 23^

Patients with TRS had an earlier onset of disease when compared with the treatment-responsive group, and early onset was a predictor of TRS. These findings are consistent with a recent study describing younger age as the strongest predictor of treatment resistance and stating that the risk to develop TRS continues to decrease throughout adulthood.^19^ Previously, Martin & Mowry,^24^ as well as Lally et al.,^25^ found a similar association between TRS and younger onset of disease. It is important to point out that younger onset is also associated with better clozapine response, and initiating clozapine promptly after TRS identification may increase clozapine effectiveness. This corroborates the hypothesis that clozapine should be initiated as early as possible in patients with TRS, in order to reduce adverse outcomes.^26 , 27^

As expected, clozapine use was significantly higher in TRS than in the treatment-responsive group. Many studies have demonstrated clozapine superiority in reducing positive and negative symptoms, as well as antipsychotic discontinuation.^12 , 16 , 28^ Despite these benefits, many obstacles for clozapine prescription remain, and a study in Brazil has described difficult access, poor adhesion to blood count routine, fear of side effects and little knowledge about the medication on the part of clinicians as the main reasons for low clozapine use.^29^ Even when clozapine is prescribed, there tends to be a delay in its initiation, leading to more severe symptoms, lower clozapine response and more electroconvulsive therapy indication.^30 , 31^ In the present study, just a small percentage of the patients with TRS did not use clozapine, because of intolerable side effects or no adhesion to routine blood tests.

The smoking rate was higher in the treatment-responsive than in the TRS group. The probable hypothesis for this is a “self-medication” phenomenon: some studies have suggested that patients with schizophrenia have a reduced density of hippocampal nicotine receptors, leading to cognitive deficits; the use of typical antipsychotics, more common among treatment-responsive individuals, may exacerbate these cognitive impairments.^32 , 33^ Hence, smoking might be a “self-medication” strategy to mitigate these impairments. However, smoking rates were lower in the present study than in other Brazilian samples of patients with schizophrenia.^34^ In another study performed at a tertiary center, Cerazetto et al.^17^ also demonstrated a low smoking prevalence in Brazilian samples, yet higher than the one found in the present study. This may indicate that information about smoking habits is not being properly recorded on patients’ charts, leading to a global underestimation of its prevalence in both groups.

APP and the use of benzodiazepines and antiepileptics were also more prevalent in TRS than among treatment-responsive patients. A recent review has stated that APP is not superior to monotherapy, except in the group of partially responsive clozapine users, which includes patients with TRS.^35^ However, many treatment-responsive individuals may be prescribed more than one antipsychotic, because of no response to monotherapy, intolerance, or incomplete cross-titration process.^36^ The findings in the present study diverge from those of Stroup et al.,^16^ who observed that clozapine use reduced APP prescription. In clinical practice, benzodiazepines are frequently used for agitation, aggressive behavior, insomnia, or anxiety, but also as add-on strategies for TRS with residual symptoms.^35^ Similarly to the findings of the present study, Wimberley et al. evidenced that benzodiazepine use was significantly associated with TRS.^8^ Antiepileptics are also add-on strategies to antipsychotic prescription, usually for reducing aggression, stabilizing mood, or treating comorbid epilepsy, but they are also used in TRS to enhance clozapine effectiveness or treat clozapine-induced seizures.^37^ All these findings are in accordance with a review by Correll et al.,^38^ which associated APP with illness severity, chronicity, and treatment resistance, and also with a study by Wagner et al.,^39^ which demonstrated frequent use of valproate to enhance clozapine. The addition of a second antipsychotic, benzodiazepine or antiepileptic to the main treatment of TRS suggests symptom severity in the patient sample. Despite the use of more medications, patients with TRS remained more symptomatic, as evidenced by the higher CGI scores.

In the present study, the investigators also found higher rates of suicide attempts and multiple hospital admissions in patients with TRS when compared to the non-resistant group. These findings reinforce the idea that patients with TRS had more severe symptoms, in accordance with previous research that also demonstrated higher hospitalization rates in treatment-resistant patients.^24^ This higher severity is also corroborated by more elevated CGI scores. In other studies, previous suicide attempt was a predictor of treatment resistance, and clozapine significantly reduced rehospitalization, all-cause mortality, suicide, and self-harm behaviors in individuals with TRS.^28 , 40^

In addition, the investigators observed no differences in DUP between the groups of the present research. These findings diverge from other studies that have highlighted the role of DUP as a predictor of TRS.^7 , 9^ Interestingly, a remarkably long period before treatment onset was noticed in the present sample, with a mean DUP of more than a year in both groups. This surprisingly long DUP may be related to the elevated TRS ratio in the sample. Despite progresses in mental health care, delays in seeking medical services after a first episode of psychosis are still a reality in some Brazilian regions, due to lack of knowledge, social or cultural reasons. In addition, the difficult access to mental health services may have contributed to longer DUP among our patients, especially those who live far from larger cities or have socioeconomic disadvantages.

It is crucial to highlight that TRS causes major financial and social burden for both health systems and individuals, and that this impact may be even more prominent in developing countries, which have limited resources for healthcare.^4^ Just a few studies have addressed TRS characteristics in the developing world. In fact, to our knowledge, this is the first study to investigate epidemiological factors related to TRS in the Northeast region of Brazil. Considering Brazil’s continental dimensions, differences in the epidemiological profile of schizophrenia across regions may exist, and understanding these disparities may help improve patient care.

## Conclusions

In the present study, being married was a protector, while younger age at disease onset was a predictor of TRS. We also demonstrated that patients with TRS had more severe symptoms, despite the higher use of clozapine and other medications. Clinicians should be more vigilant in individuals with these characteristics, to identify TRS at an early stage and initiate clozapine promptly after treatment resistance identification.

### Limitations

This study has several limitations. Data on symptoms, medication and compliance may have been affected by information bias. Because the investigators collected information from the patients’ records, some data were unavailable or missing. Also, some clinical information may not have been recorded by the assistant physicians and is consequently not reported on charts. Patients were selected from a referral center responsible for treating severe cases, hence the individuals included may have been subjected to selection bias. Finally, the retrospective and observational design of the study makes it subject to some confounding factors: first, it was not possible to confirm the diagnosis of schizophrenia using structured interviews, once the researchers did not have direct contact with the patients (rather, only with their records); second, because of the retrospective nature of the study, it was not possible to establish direct causes for the phenomena described in the article, and no interventions were investigated; third, our study was conducted at one medical center located in one of the five Brazilian regions, hence the generalization of results to other populations requires caution.

## Trends in Psychiatry and Psychotherapy - Online-Only Supplementary Material


