# Supplementary material for: Prevalence and predictors of treatment-resistant schizophrenia in a tertiary hospital in Northeast Brazil
Source: Trends Psychiatry Psychother. 2021 Dec 10;43(4):270–7. doi: 10.47626/2237-6089-2020-0151 (PMC8835382; doi:10.47626/2237-6089-2020-0151)
Supplement: Supplementary file 1 [file 2238-0019-trends-43-04-0270-suppl01.pdf]

http://dx.doi.org/10.47626/2237-6089-2020-0151

Table S1 - Clinical and psychiatric comorbidities of patients with TRS and treatment-responsive patients

	Treatment-resistant		
	**Yes (n = 155)**	**No (n = 50)**	**p**
Depression			
Yes	12 (7.7)	3 (6.0)	0.618
No	143 (92.3)	47 (94)	
Anxiety			
Yes	22 (14.2)	7 (14)	0.973
No	133 (85.8)	43 (86)	
Obsessive-compulsive disorder			
Yes	38 (24.5)	10 (20)	0.512
No	117 (75.5)	40 (80)	
Substance use			
Yes	24 (15.5)	12 (24.5)	0.149
No	131 (84.5)	38 76)	
Current smoking			
Yes	17 (11)	11 (22)	0.048
No	138 (89)	39 (78)	
Diabetes			
Yes	23 (14.8)	7 (14)	0.884
No	132 (85.2)	43 (76)	

Data presented as n (%).
Pearson’s chi-square test was used.
